# Creep damage model of rock with varying-parameter under the step loading and unloading conditions

**DOI:** 10.1038/s41598-021-03539-7

**Published:** 2021-12-15

**Authors:** Guanghe Li, Yanting Wang, Dong Wang, Xiaoxu Yang, Laigui Wang, Yanfei Li, Shipeng Zhang

**Affiliations:** 1 College of Mining, Liaoning Technical University, Fuxin, 123000 China; 2 College of Mechanics Engineering, Liaoning Technical University, Fuxin, 123000 China; 3 Shenhua Bor Xil Energy Co. Ltd., Hulun Buir, 021008 China

**Keywords:** Civil engineering, Natural hazards, Petrology

## Abstract

The creep characteristics of rock under step loading and unloading conditions were investigated in this study. Based on the generalized Burgers model, the total strain of rock was decomposed into elastic, viscoelastic, varying-parameter viscoelastic, and viscoplastic strains considering the damage. The four strains were connected in series to establish a new varying-parameter creep damage model that can characterize the creep characteristics of rock under step loading and unloading conditions as well as identify and verify the model parameters. The study results showed that the varying-parameter creep damage model could better describe the creep characteristics of rock under step loading and unloading conditions, significantly the non-linear both the strain and time of attenuation creep and accelerated creep. The model fitting curve was highly consistent with the experimental data, and the correlation coefficient *R*^2^ was greater than 0.98, which thoroughly verified the accuracy and rationality of the model. These findings can provide theoretical support for analyzing the deformation and long-term stability of rock and soil.

## Introduction

Rock creep has been a prominent research hotspot for experts and scholars in geotechnical engineering. Rock creep is closely related to rock and soil deformation and long-term stability^1^. In the development process of slope excavation and tunnel excavation, the surrounding rock is typically in loading and unloading states. The loading and unloading effects seriously weakens the strength of the rock mass and induces secondary disasters^2,3^. Therefore, understanding the rock creep model under step loading and unloading conditions is theoretically significant and practically valuable for slope engineering design, landslide warning, and tunnel deformation prevention.

Scholars have achieved fruitful advancements in studying rock creep models, which are mainly categorized into empirical and component combination models^4–6^. Empirical models are based on experimental phenomena and laws^7–10^; component combination models are based on essential components, such as elasticity, viscosity, and plasticity, and are formed by series and parallel methods^11,12^. Wang et al.^13^ established a six-element composite creep model by analyzing the loading and unloading creep curves of limestone. Song et al.^14^ used saturated red sandstone as the step loading and unloading creep test object and constructed a freeze–thaw-damage creep model. Deng et al.^15^ utilized Q3 loess as a research object, introduced a fractional element model based on the Nishihara model, and deduced the constitutive equation of rock under the cyclic loading and unloading conditions. Yang et al.^16^ conducted graded loading and unloading uniaxial creep tests on sandstone and established a creep damage model. Based on the triaxial cyclic loading and unloading creep phenomenon of rock, Yang et al.^17^ separated rock strain by viscoelastic-plasticity and constructed a creep model considering the damage. Wang et al.^18^ conducted triaxial compression tests and unloading confining pressure tests to determine the mechanical properties of sandstone under loading and unloading conditions. Despite these efforts, no study has investigated the rock creep model under step loading and unloading conditions, which considers the damage of the accelerated creep stage and the nonlinear function of the viscosity coefficient and time.

Based on previous research results, this study utilized the sandstone of a tunnel floor as the test object, introduced the time-dependent viscosity coefficient into the viscoelastic body, and introduced the damage variable into the viscoelastic body. The total strain of the rock was decomposed into elastic, viscoelastic, variable parameter viscoelastic, and viscoplastic strains considering the damage. The four strains were connected in series to establish a new variable-parameter creep damage model that can characterize the creep characteristics of rock at all stages under hierarchical loading and unloading conditions. One-dimensional and three-dimensional constitutive equations were derived, and the creep damage equation under hierarchical loading and unloading conditions was obtained according to the superposition principle. Finally, a simple least-squares method was used for parameter identification and verification.

## Analysis of sandstone creep test data under step loading and unloading conditions

**Figure 1:**
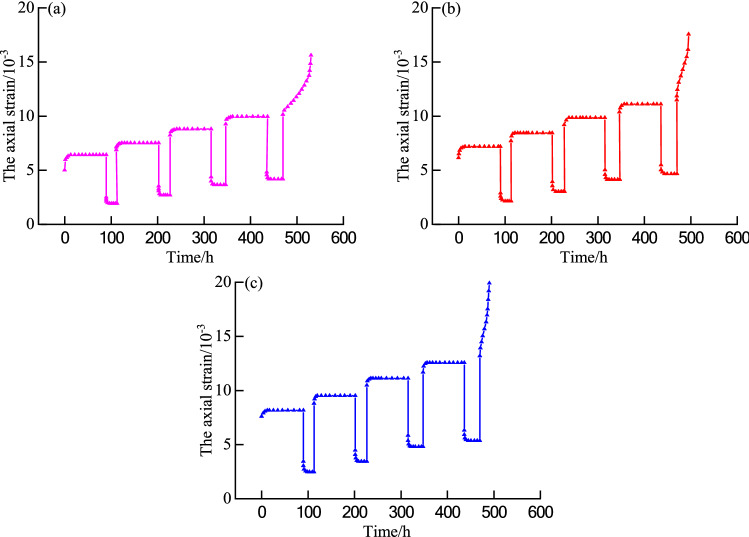
Sandstone creep curve under step loading and unloading conditions. **(a)** 4 MPa, **(b)** 8 MPa, **(c)** 12 MPa.

Yang et al.^19^ utilized the sandstone of a tunnel floor as the test object: the sample size was Φ50 mm × 100 mm, and the RLW-2000 triaxial rheological test system was used to conduct a step loading and unloading creep test on the sandstone sample. The confining pressures were 4 MPa, 8 MPa, and 12 MPa, respectively. The levels of deviator stress during the test are shown in Table 1, and the creep test curve of graded loading and unloading sandstone is shown in Fig. 1.

**Table 1:** The levels of deviator stress during the test.

Confining pressure (MPa)	Deviator stress (MPa)				
	Level 1	Level 2	Level 3	Level 4	Level 5
4	11.99	14.99	17.99	20.97	23.98
8	16.61	20.76	24.91	29.06	33.22
12	22.48	28.11	33.73	39.35	44.97

Figure 1 indicates that instantaneous elastic strain occurs initially at low-stress levels (level 1 to level 4). As the loading time increases, the axial strain rate of sandstone gradually decreases, and the axial strain increases nonlinearly, all tending to a particular value. With an increase in the unloading time, the axial strain rate and axial strain of the sandstone gradually decrease, and both tend to a particular value. The sandstone sample exhibits attenuation and constant velocity creep stages under the step loading and unloading conditions. At high-stress levels (level 5), the sandstone has a nonlinear accelerated creep phase. In summary, the creep stages of sandstone under step loading and unloading conditions include attenuation creep, constant velocity creep, and accelerated creep stages.

## Establishment of the varying-parameter creep damage model

**Figure 2:**
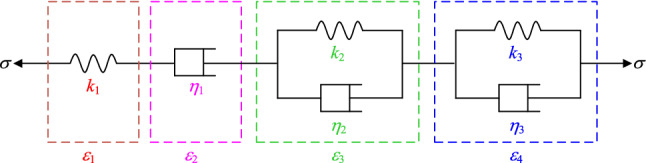
The generalized Burgers model.

**Figure 3:**
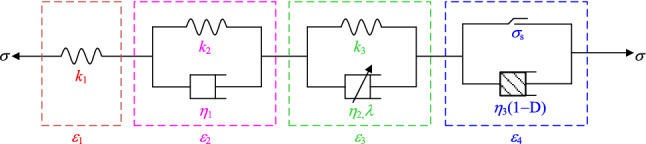
The varying-parameter creep damage model.

The classic Nishihara and Burgers creep models can describe the deformation characteristics of rock attenuation creep and constant velocity creep. However, the models cannot describe the nonlinear law of strain and time in the accelerated creep stage. Therefore, to accurately describe the full-stage creep characteristics of the rock under step loading and unloading conditions, a varying-parameter creep damage model is established based on the generalized Burgers model^20^ (as shown in Fig. 2). The new model can consider the immense creep damage of the rock during the acceleration stage^21^, and the viscosity coefficient, which should be a nonlinear function, is a power function related to time^22^. The new model is illustrated in Fig. 3.

### Elastomer

Lin et al.^23^ established a one-dimensional creep equation of an elastometer,

1 $$ \varepsilon_{1} = \frac{\sigma }{{k_{1} }}, $$

where σ is the stress of the elastometer, *k*_1_ is the elastic modulus of the elastometer, and *ε*_1_ is the strain of the elastometer.

The creep equation of an elastometer under three-dimensional stress can be written as follows^24^:

2 $$ \left\{ {\begin{array}{*{20}c} {\varepsilon_{ij}^{1} = \frac{3(1 - 2v)}{{2k_{1} }}S_{ij} + \frac{2(1 + v)}{{3k_{1} }}\sigma_{m} \delta_{ij} } \\ { = \frac{{S_{ij} }}{{2G_{1} }} + \frac{{\sigma_{m} \delta_{ij} }}{3K}} \\ \end{array} } \right., $$

where *v* is the Poisson’s ratio of rock, *S*_*ij*_ is the deviation stress tensor, *σ*_m_ is the average stress, *δ*_*ij*_ is the Kronecker tensor, *G*_1_ is the shear modulus of the elastometer, and *K* is the bulk modulus of the elastometer.

The deviatoric stress tensor in the principal stress space can be expressed as

3 $$ S_{ij} { = }\sigma_{ij} - \sigma_{m} \delta_{ij} . $$

### Viscoelastic body

The viscoelastic body is also known as a Kelvin’s material, and its constitutive equation is^25^

4 $$ {{\sigma = \eta }}_{1} \frac{{d{\upvarepsilon }_{2} }}{{d{\text{t}}}} + {\text{k}}_{2} {\upvarepsilon }_{2} , $$

where *ε*_2_ is the strain of the Kelvin’s material, *k*_2_ is the elastic modulus of the Kelvin’s material, and *η*_1_ is the viscosity coefficient of the viscoelastic body (Kelvin’s material).

A one-dimensional creep equation of the viscoelastic body is established as follows^26^:

5 $$ \varepsilon_{2} = \frac{\sigma }{{k_{2} }}\left[ {1 - \exp \left( - \frac{{k_{2} }}{{\eta_{1} }}t\right)} \right]. $$

By extending to the three-dimensional space analysis, the three-dimensional creep equation of the viscoelastic body can be obtained as^27^

6 $$ \varepsilon_{ij}^{2} = \frac{{S_{ij} }}{{2G_{2} }}\left[ {1 - \exp \left( - \frac{{G_{2} }}{{\eta_{1} }}t\right)} \right], $$

where *G*_2_ is the shear modulus of the viscoelastic body.

Assume that the unloading occurs at *t*_0_, Eq. (4) can be determined using

7 $$ \varepsilon_{2} = A\exp \left( { - \frac{{k_{2} }}{{\eta_{1} }}t_{0} } \right), $$

where *A* is the integral constant.

When *t* = *t*_0,_ Eq. (5) can be written as follows:

8 $$ \varepsilon_{2} = \frac{\sigma }{{k_{2} }}\left[ {1 - \exp \left( - \frac{{k_{2} }}{{\eta_{1} }}t_{0} \right)} \right]. $$

Based on Eq. (7) and Eq. (8), the integral constant *A* is given by

9 $$ A = \frac{\sigma }{{k_{2} }}\left[ {1 - \exp\left ( - \frac{{k_{2} }}{{\eta_{1} }}t_{0} \right)} \right]\exp\left (\frac{{k_{2} }}{{\eta_{1} }}t_{0}\right ). $$

Based on Eq. (7) and Eq. (9), the one-dimensional creep equation of the viscoelastic body under unloading conditions can be established as

10 $$ \varepsilon_{n1} = \frac{\sigma }{{k_{2} }}\left[ {1 - \exp\left ( - \frac{{k_{2} }}{{\eta_{1} }}t_{0}\right )} \right]\exp \left[ {\frac{{k_{2} }}{{\eta_{1} }}(t_{0} - t)} \right]. $$

By extending to the three-dimensional space analysis, the three-dimensional creep equation of the viscoelastic body under unloading conditions can be obtained as

11 $$ \varepsilon_{ij}^{n1} = \frac{{S_{ij} }}{{2G_{2} }}\left[ {1 - \exp \left( - \frac{{G_{2} }}{{\eta_{1} }}t_{0}\right )} \right]\exp \left[ {\frac{{G_{2} }}{{\eta_{1} }}(t_{0} - t)} \right]. $$

### Varying-parameter viscoelastic body

Zhang et al.^28^ assumed that the viscosity coefficient is a power function related to time; function *η*_2_(*t*) of the viscosity coefficient related to time is given as follows:

12 $$ \eta_{{2}} (t) = \eta_{{{20}}} t^{1 - \lambda } , $$

where *η*_20_ is the initial viscosity coefficient of the varying-parameter viscoelastic body, and *λ* is a constant.

According to the one-dimensional constitutive equation of a Kelvin’s material, a one-dimensional creep equation of the varying-parameter viscoelastic body is established as follows:

13 $$ \sigma = k_{3} \varepsilon_{3} + \eta_{{2}} (t)\dot{\varepsilon }_{3} = k_{3} \varepsilon_{3} + \eta_{{{20}}} t^{1 - \lambda } \dot{\varepsilon }_{3} , $$

where *ε*_3_ is the strain of the varying-parameter viscoelastic body, and *k*_3_ is the elastic modulus.

A one-dimensional creep equation of the varying-parameter viscoelastic body is established by integrating Eq. (13) as follows:

14 $$ \varepsilon_{3} = \frac{\sigma }{{k_{3} }}\left[ {1 - \exp \left ( - \frac{{k_{3} }}{{\eta_{{{20}}} \lambda }}t^{\lambda }\right )} \right]. $$

By extending to the three-dimensional space analysis, the three-dimensional creep equation of the varying-parameter viscoelastic body can be obtained as

15 $$ \varepsilon_{ij}^{3} = \frac{{S_{ij} }}{{2G_{3} }}\left[ {1 - \exp\left ( - \frac{{G_{3} }}{{\eta_{{{20}}} \lambda }}t^{\lambda }\right )} \right], $$

where *G*_3_ is the shear modulus of the varying-parameter viscoelastic body.

If *t*_0_ is the unloading time, Eq. (13) can be written as follows:

16 $$ \dot{\varepsilon }_{3} + \frac{{k_{3} }}{{\eta_{{{20}}} t_{0}^{1 - \lambda } }}\varepsilon_{3} = 0. $$

Integrate Eq. (16) as follows:

17 $$ \varepsilon_{3} = B\exp \left( - \frac{{k_{3} }}{{\eta_{{{20}}} t_{0}^{1 - \lambda } }}t\right), $$

where *B* is the integral constant.

When *t* = *t*_0_, Eq. (14) can be written as follows:

18 $$ \varepsilon_{3} = \frac{\sigma }{{k_{3} }}\left[ {1 - \exp\left ( - \frac{{k_{3} }}{{\eta_{{{20}}} \lambda }}t_{0}^{\lambda }\right )} \right]. $$

Based on Eq. (17) and Eq. (18), the integral constant *B* can be written as follows:

19 $$ B = \frac{\sigma }{{k_{3} }}\left[ {1 - \exp\left ( - \frac{{k_{3} }}{{\eta_{{{20}}} \lambda }}t_{0}^{\lambda }\right )} \right]\exp\left (\frac{{k_{3} }}{{\eta_{{{20}}} t_{0}^{1 - \lambda } }}t_{0}\right ). $$

Based on Eq. (17) and Eq. (19), the one-dimensional creep equation of the varying-parameter viscoelastic body under unloading conditions can be established as

20 $$ \varepsilon_{n2} = \frac{\sigma }{{k_{3} }}\left[ {1 - \exp\left ( - \frac{{k_{3} }}{{\eta_{{{20}}} \lambda }}t_{0}^{\lambda }\right )} \right]\exp \left[ {\frac{{k_{3} }}{{\eta_{{{20}}} t_{0}^{1 - \lambda } }}(t_{0} - t)} \right]. $$

By extending to the three-dimensional space analysis, the three-dimensional creep equation of the varying-parameter viscoelastic body under unloading conditions can be obtained as

21 $$ \varepsilon_{{_{ij} }}^{n2} = \frac{{S_{ij} }}{{2G_{3} }}\left[ {1 - \exp\left (\frac{{ - G_{3} }}{{\eta_{{{20}}} \lambda }}t_{0}^{\lambda } \right)} \right]\exp \left[ {\frac{{G_{3} }}{{\eta_{{{20}}} t_{0}^{1 - \lambda } }}(t_{0} - t)} \right]. $$

### Damaged viscoplastic body

The author introduces the damage variable *D* to describe the creep damage degradation of the viscosity coefficient and constructs a viscoplastic body considering the damage. Its one-dimensional constitutive equation can be written as follows:

22 $$ \sigma = \eta_{{3}} (t)\dot{\varepsilon }_{4} , $$

where $\dot{\varepsilon }_{4}$ is the strain rate of the damaged viscoplastic body, and *η*_3_(*t*) is the function of the viscosity coefficient related to time.

Based on the results of numerous rock creep damage tests, the damage variable *D* took the form of a negative exponential function related to time during rock creep^29–32^. In this study, the damage variable is reduced to Eq. (23).

23 $$ D = 1 - e^{ - \alpha t} ,(0 < D < 1), $$

where *α* is the coefficient related to the properties of rock materials.

Based on $\eta_{{3}} (t) = \eta_{{3}} (1 - D) = \eta_{{3}} e^{ - \alpha t}$ and Eq. (22), the one-dimensional creep equation of the damaged viscoplastic body can be written as follows:

24 $$ \varepsilon_{4} = \frac{\sigma }{{\eta_{{3}} \alpha }}e^{\alpha t} , $$

where *ε*_4_ is the strain of the damaged viscoplastic body, and *η*_3_ is the initial viscosity coefficient of the damaged viscoplastic body.

The three-dimensional creep equation of the damaged viscoplastic body can be obtained as:

25 $$ \left\{ {\begin{array}{*{20}c} {\varepsilon_{{_{ij} }}^{4} = \frac{1}{{\eta_{{3}} }}\left\langle {\phi \left[ {\frac{F}{{F_{0} }}} \right]} \right\rangle \frac{\partial Q}{{\partial \sigma_{ij} }}t} \\ {\left\langle {\phi \left[ {\frac{F}{{F_{0} }}} \right]} \right\rangle = \left\{ {\begin{array}{*{20}c} {\begin{array}{*{20}c} 0 & {(F < 0)} \\ \end{array} } \\ {\begin{array}{*{20}c} {\phi \left[ {\frac{F}{{F_{0} }}} \right]} & {(F \ge 0)} \\ \end{array} } \\ \end{array} } \right.} \\ \end{array} } \right., $$

where *F*_0_ is the initial reference value of the rock yield function, *F* is the rock yield function, *Q* is the plastic potential function, and $\phi ( \cdot )$ is the power function form.

When *F* ≥ 0, based on Eq. (25) and associated flow standards, the following relationship can be obtained:

26 $$ \varepsilon_{{_{ij} }}^{4} = \frac{1}{{\eta_{{3}} }}\left[ {\frac{F}{{F_{0} }}} \right]\frac{\partial F}{{\partial \sigma_{ij} }}t, $$

where

27 $$ F = \sqrt {J_{2} } - \sigma_{s} /\sqrt 3 , $$

and *σ*_s_ is the yield strength of the rock.

Under the conventional triaxial compression test conditions (σ_2_ = σ_3_), *J*_2_ can be obtained as

28 $$ \sqrt {J_{2} } = \frac{1}{\sqrt 3 }(\sigma_{1} - \sigma_{3} ). $$

Assume that the initial reference value of the rock yield function *F*_0_ = 1, then the three-dimensional creep equation of the damaged viscoplastic body can be written as follows:

29 $$ \varepsilon_{{_{ij} }}^{4} = \frac{{\sigma_{1} - \sigma_{3} }}{{3\eta_{{3}} }}e^{\alpha t} t. $$

### Establishment of the varying-parameter creep damage model

According to the series character of components, the following relationship can be obtained:

30 $$ \left\{ {\begin{array}{*{20}c} {\varepsilon = \varepsilon_{1} + \varepsilon_{2} + \varepsilon_{3} + \varepsilon_{4} } \\ {\varepsilon_{ij} = \varepsilon_{ij}^{1} + \varepsilon_{ij}^{2} + \varepsilon_{ij}^{3} + \varepsilon_{ij}^{4} } \\ {\varepsilon_{n} = \varepsilon_{n1} + \varepsilon_{n2} } \\ {\varepsilon_{ij}^{n} = \varepsilon_{ij}^{n1} + \varepsilon_{ij}^{n2} } \\ \end{array} } \right.. $$

In the conventional triaxial compression creep test, the second principal stress $\sigma_{2} = \sigma_{3}$, then

31 $$ \left\{ {\begin{array}{*{20}c} {\sigma_{m} = \frac{1}{3}(\sigma_{1} + \sigma_{{2}} + \sigma_{3} ) = \frac{1}{3}(\sigma_{1} + 2\sigma_{3} )} \\ {S_{{{11}}} = \sigma_{1} - \sigma_{m} = \frac{2}{3}(\sigma_{1} - \sigma_{3} )} \\ \end{array} } \right., $$

where *σ*_1_ is axial compression, *σ*_2_ is confinement pressure, *σ*_3_ is confinement pressure, and *S*_11_ is axial deviatoric stress tensor.

Based on Eqs. (1), (5), (14), (24), and (30), the one-dimensional creep equation of the varying-parameter creep damage equation under loading conditions can be obtained as

32 $$ \varepsilon = \left\{ {\begin{array}{*{20}c} {\begin{array}{*{20}c} \begin{gathered} \frac{\sigma }{{k_{1} }} + \frac{\sigma }{{k_{2} }}\left[ {1 - \exp \left( - \frac{{k_{2} }}{{\eta_{1} }}t \right)} \right] + \hfill \\ \frac{\sigma }{{k_{3} }}\left[ {1 - \exp \left ( - \frac{{k_{3} }}{{\eta_{{{20}}} \lambda }}t^{\lambda }\right )} \right] \hfill \\ \end{gathered} & {\sigma < \sigma_{s} } \\ \end{array} } \\ {\begin{array}{*{20}c} \begin{gathered} \frac{\sigma }{{k_{1} }} + \frac{\sigma }{{k_{2} }}\left[ {1 - \exp \left( - \frac{{k_{2} }}{{\eta_{1} }}t \right)} \right] + \\ \frac{\sigma }{{k_{3} }}\left[ {1 - \exp\left (\frac{{ - k_{3} }}{{\eta_{{{20}}} \lambda }}t^{\lambda }\right )} \right] + \frac{{\sigma - \sigma_{s} }}{{\eta_{{3}} \alpha }}e^{\alpha t} \\ \end{gathered} & {\sigma \ge \sigma_{s} } \\ \end{array} } \\ \end{array} } \right.. $$

Based on Eqs. (2), (6), (15), (29), (30), and (31), the three-dimensional creep equation of the varying-parameter creep damage equation under loading conditions can be obtained as

33 $$ \varepsilon_{{{11}}} = \left\{ {\begin{array}{*{20}c} {\begin{array}{*{20}c} \begin{gathered} \frac{{\sigma_{1} - \sigma_{3} }}{{3G_{1} }} + \frac{{\sigma_{1} + 2\sigma_{3} }}{9K} + \frac{{\sigma_{1} - \sigma_{3} }}{{3G_{2} }}\left[ {1 - \exp \left( - \frac{{G_{2} }}{{\eta_{1} }}t \right)} \right]  \\ +\frac{{\sigma_{1} - \sigma_{3} }}{{3G_{3} }}\left[ {1 - \exp \left( - \frac{{G_{3} }}{{\eta_{{{20}}} \lambda }}t^{\lambda } \right )} \right] \\ \end{gathered} & {\sigma < \sigma_{s} } \\ \end{array} } \\ {\begin{array}{*{20}c} \begin{gathered} \frac{{\sigma_{1} - \sigma_{3} }}{{3G_{1} }} + \frac{{\sigma_{1} + 2\sigma_{3} }}{9K} + \frac{{\sigma_{1} - \sigma_{3} }}{{3G_{2} }}\left[ {1 - \exp\left ( - \frac{{G_{2} }}{{\eta_{1} }}t \right)} \right]  \\ + \frac{{\sigma_{1} - \sigma_{3} }}{{3G_{3} }}\left[ {1 - \exp \left (\frac{{ - G_{3} }}{{\eta_{{{20}}} \lambda }}t^{\lambda }\right )} \right] + \frac{{\sigma_{1} - \sigma_{3} - \sigma_{s} }}{{3\eta_{{3}} }}e^{\alpha t} t \\ \end{gathered} & {\sigma \ge \sigma_{s} } \\ \end{array} } \\ \end{array} } \right.. $$

The viscoelastic strain cannot be recovered under unloading conditions. Based on Eqs. (10), (20), and (30), the one-dimensional creep equation of the varying-parameter creep damage equation under unloading conditions can be obtained as

34 $$ \begin{aligned} \varepsilon_{n} &= \frac{\sigma }{{k_{2} }}\left[ {1 - \exp \left ( - \frac{{k_{2} }}{{\eta_{1} }}t_{0} \right)} \right]\exp \left[ {\frac{{k_{2} }}{{\eta_{1} }}(t_{0} - t)} \right] \\ &\quad +  \frac{\sigma }{{k_{3} }}\left[ {1 - \exp\left (\frac{{ - k_{3} }}{{\eta_{{{20}}} \lambda }}t_{0}^{\lambda }\right )} \right]\exp \left[ {\frac{{k_{3} }}{{\eta_{{{20}}} t_{0}^{1 - \lambda } }}(t_{0} - t)} \right]. \\ \end{aligned} $$

Based on Eqs. (11), (21), (30), and (31), the three-dimensional creep equation of the varying-parameter creep damage equation under unloading conditions can be obtained as

35 $$ \begin{gathered} \varepsilon_{11}^{n} = \frac{{\sigma_{1} - \sigma_{3} }}{{3G_{2} }}\left[ {1 - \exp \left( - \frac{{G_{2} }}{{\eta_{1} }}t_{0} \right)} \right]\exp \left[ {\frac{{G_{2} }}{{\eta_{1} }}(t_{0} - t)} \right] + \\ \frac{{\sigma_{1} - \sigma_{3} }}{{3G_{3} }}\left[ {1 - \exp \left(\frac{{ - G_{3} }}{{\eta_{{{20}}} \lambda }}t_{0}^{\lambda } \right)} \right]\exp \left[ {\frac{{G_{3} }}{{\eta_{{{20}}} t_{0}^{1 - \lambda } }}(t_{0} - t)} \right]. \\ \end{gathered} $$

## Model parameter identification and validation

To verify the accuracy and rationality of the variable parameter creep damage model, the author used MATLAB to identify the parameters of the varying-parameter creep damage model based on the sandstone creep test data under step loading and unloading conditions. To simplify the parameter identification process, letters are used to replace the complicated items in the theoretical model; the following relationship can be obtained:

36 $$ \left\{ {\begin{array}{*{20}c} {A = \frac{{\sigma_{1} - \sigma_{3} }}{{3G_{1} }} + \frac{{\sigma_{1} + 2\sigma_{3} }}{9K}} \\ {B = \frac{{G_{2} }}{{\eta_{1} }}} \\ {C = \frac{{G_{3} }}{{\eta_{{{20}}} \lambda }}} \\ {D = \frac{{G_{3} }}{{\eta_{{{20}}} }}} \\ \end{array} } \right.. $$

Based on Eqs. (33) and (36), we have

37 $$ \varepsilon_{11} = \left\{ {\begin{array}{*{20}c} {\begin{array}{*{20}c} \begin{gathered} A + \frac{{\sigma_{1} - \sigma_{3} }}{{3G_{2} }}\left[ {1 - \exp ( - Bt)} \right] + \hfill \\ \frac{{\sigma_{1} - \sigma_{3} }}{{3G_{3} }}\left[ {1 - \exp ( - Ct^{\lambda } )} \right] \hfill \\ \end{gathered} & {\sigma < \sigma_{s} } \\ \end{array} } \\ {\begin{array}{*{20}c} \begin{gathered} A + \frac{{\sigma_{1} - \sigma_{3} }}{{3G_{2} }}\left[ {1 - \exp ( - Bt)} \right] + \hfill \\ \frac{{\sigma_{1} - \sigma_{3} }}{{3G_{3} }}\left[ {1 - \exp ( - Ct^{\lambda } )} \right] + \hfill \\ \frac{{\sigma_{1} - \sigma_{3} - \sigma_{s} }}{{3\eta_{{3}} }}e^{\alpha t} t \hfill \\ \end{gathered} & {\sigma \ge \sigma_{s} } \\ \end{array} } \\ \end{array} } \right.. $$

Based on Eqs. (35) and (36), we have

38 $$ \varepsilon_{{{11}}}^{n} = \frac{{\sigma_{1} - \sigma_{3} }}{{3G_{2} }}\left[ {1 - \exp ( - Bt_{0} )} \right]\exp \left[ {B(t_{0} - t)} \right] + \frac{{\sigma_{1} - \sigma_{3} }}{{3G_{3} }}\left[ {1 - \exp ( - Ct_{0}^{\lambda } )} \right]\exp \left[ {\frac{D}{{t_{0}^{1 - \lambda } }}(t_{0} - t)} \right]. $$

**Figure 4:**
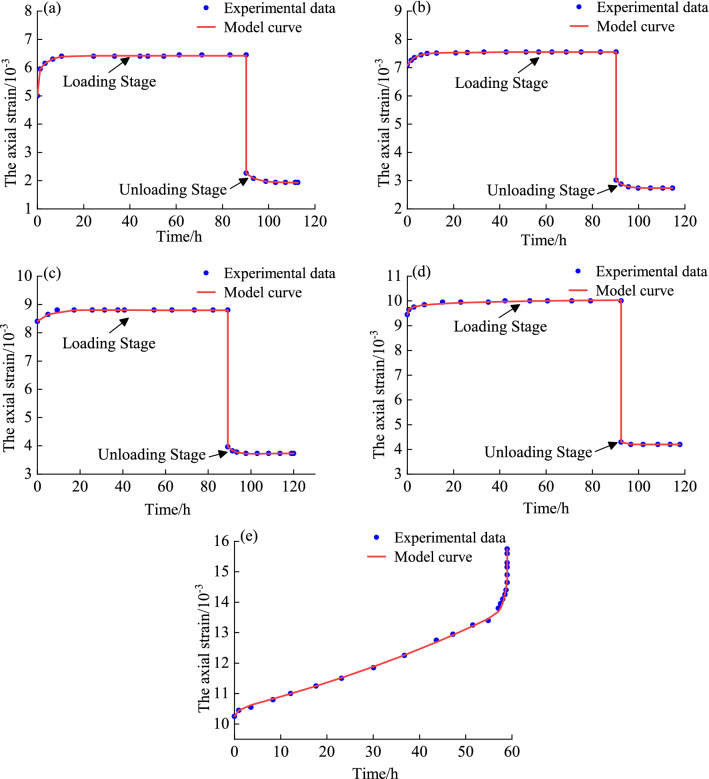
Comparative analysis of model curve and test data when the confining pressure is 4 MPa. **(a)** 11.99 MPa, **(b)** 14.99 MPa, **(c)** 17.99 MPa, **(d)** 20.97 MPa, **(e)** 23.98 MPa.

After solving A, B, C, and D, the parameters in the model were solved and inputted into the varying-parameter creep damage model to obtain the correlation coefficient between the model curve and the test curve. The results are shown in Tables 2 and 3. Owing to many images, only the model curve and test data when the confining pressure is 4 MPa are listed (as shown in Fig. 4).

**Table 2:** Model parameter identification results in the loading condition.

Confining pressure (MPa)	Deviator stress (MPa)	*G*_1_ (MPa)	*K *(MPa)	*G*_2_ (MPa)	*η*_1_ (MPa·h)	*G*_3_ (MPa)	*λ*	*η*_20_ (MPa·h)	*η*_3_ (MPa·h)	*α*	*R*^2^
4	11.99	2.1897	0.8397	3.1868	0.8560	4.5325	1.1306	20.6966	–	–	0.9985
	14.99	1.8054	0.6923	8.5186	29.3340	5.9157	0.0703	41.2052	–	–	0.9999
	17.99	1.7483	0.6704	9.5215	54.3465	9.4636	0.0197	526.1619	–	–	0.9998
	20.97	1.7528	0.6721	24.6182	8.5060	15.0339	0.7525	111.0539	–	–	0.9891
	23.98	1.7970	0.6891	0.7516	98.8947	7.0724	0.7844	563.5199	0.9983	11.0271	0.9940
8	16.61	2.8090	1.0771	5.4367	20.0173	3.2480	0.0734	67.9943	–	–	0.9955
	20.76	2.4951	0.9568	9.6333	27.2050	5.6150	1.3492	5945.3221	–	–	0.9966
	24.91	2.3964	0.9189	13.0854	1.7946	7.2915	0.0517	2727.9462	–	–	0.9977
	29.06	2.3662	0.9074	26.9699	190.1968	21.7746	0.4552	51.7417	–	–	0.9989
	33.22	2.3836	0.9140	14.5633	3.4097	0.1586	0.8631	41.7628	3.6402	101.5259	0.9959
12	22.48	3.5610	1.3655	5.4102	1.1492	5.4725	1.0255	28.4761	–	–	0.9988
	28.11	3.2033	1.2283	16.4465	26.5952	9.3529	0.2514	38.4888	–	–	0.9983
	33.73	3.0032	1.1516	16.1156	33.9204	38.9416	3.6305	11.6831	–	–	0.9999
	39.35	2.9572	1.1340	22.6859	17.2281	9.7773	0.3595	155.3223	–	–	0.9967
	44.97	2.8691	1.1001	12.6050	10.7597	2.6264	1.5966	143.0431	1.1562	170.0202	0.9991

**Table 3:** Model parameter identification results in the unloading condition.

Confining pressure (MPa)	Time period	*G*_2_ (MPa)	*η*_1_ (MPa·h)	*G*_3_ (MPa)	*λ*	*η*_20_ (MPa·h)	*R*^2^
4	After level 1	2.8116	10.6995	0.0280	1.4628	0.0330	0.9985
	After level 2	5.0531	14.8730	0.1549	25.2962	0.0080	0.9990
	After level 3	12.0426	18.1450	0.5411	24.9442	0.0246	0.9939
	After level 4	3.8941	11.0090	0.2865	11.4404	0.0173	0.9849
8	After level 1	7.8248	5.0745	0.1941	1.4831	0.2162	0.9934
	After level 2	10.1660	9.3348	0.4349	13.8946	0.0445	0.9951
	After level 3	3.3233	3.2988	0.0023	4.5079	0.0008	0.9983
	After level 4	8.9977	15.6245	0.0003	27.4785	0.0001	0.9907
12	After level 1	3.2486	2.8257	0.4599	8.0931	0.0584	0.9909
	After level 2	5.9537	4.1234	0.7569	10.7438	0.0822	0.9838
	After level 3	18.2933	28.5893	0.1578	23.0808	0.0133	0.9902
	After level 4	16.8359	21.1461	0.8046	22.0334	0.0520	0.9928

It can be seen from Fig. 4 that the varying-parameter creep damage model can better simulate the three stages of rock creep Moreover, the scattered points are approximately on both sides of the fitting curve, indicating that the model can achieve results under different loading stress conditions and different lithologies. The better fit results demonstrate the correctness and applicability of the model.

As shown in Fig. 4a–d, regardless of the stage (loading or unloading), the model curve is highly consistent with the changing trend of the test data and can better describe the creep mechanical behavior of rocks. Hence, the model is also suitable for studying the law of stable creep deformation of rocks.

It can be observed from Fig. 4e that the accelerated creep phase occurs quickly, and the creep curve rises rapidly. The model can also capture the creep deformation law adequately, especially as the description of the inflection point is more accurate. The model can describe the nature of the rock strain changing drastically with time in the accelerated creep stage, which fully reflects the superiority of the established model for describing the nonlinear accelerated creep characteristics of rock.

Overall, the varying-parameter creep damage model has a better identification effect and a higher fitting accuracy under different confining pressures and deviator stresses. The model can better characterize the creep properties of rock during loading and unloading, especially the nonlinear changes of strain and time in the attenuation creep and accelerated creep stages. The fitting curve is highly consistent with the experimental data, the correlation coefficient *R*^2^ is greater than 0.98, and the parameters conform to knowledge of physics and mechanics; therefore, the accuracy and rationality of the model are thoroughly verified. consequently, the varying-parameter creep damage model developed in this study can be used to predict the creep deformation of rock under long-term loading and unloading stress.

## Conclusion

The total strain of rock is decomposed into elastic, viscoelastic, varying-parameter viscoelastic, and viscoplastic strains considering the damage. The four strains were connected in series to establish a new varying-parameter creep damage model that can characterize the creep characteristics of rock under step loading and unloading conditions. The varying-parameter creep damage model could better describe the creep characteristics of rock under step loading and unloading conditions, significantly the non-linear both the strain and time of attenuation creep and accelerated creep. The model fitting curve was highly consistent with the experimental data, and the correlation coefficient *R*^2^ was greater than 0.98, which thoroughly verified the accuracy and rationality of the model.

## Data Availability

The data used to support the findings of this study are available from the corresponding author upon request.
